# Obésité, activité physique et temps de sédentarité chez des adolescents scolarisés, âgés de 15 à 18 ans de la ville de Sfax (Tunisie)

**DOI:** 10.11604/pamj.2015.22.370.6121

**Published:** 2015-12-16

**Authors:** Sofien Regaieg, Nadia Charfi, Mouna Elleuch, Fatma Mnif, Rim Marrakchi, Sourour Yaich, Kamel Jammousi, Jamel Damak, Mohamed Abid

**Affiliations:** 1 Unité de Recherche Obésité-Syndrome Métabolique, Service d’'Endocrinologie, CHU Hédi Chaker, Sfax, Tunisie; 2 Laboratoire de Biochimie, CHU Hédi Chaker, SFAX, Tunisie; 3 Service Médecine Communautaire et d'Epidémiologie, CHU Hédi Chaker, Sfax, Tunisie

**Keywords:** Obésité, surpoids, activité physique, activité sédentaire, adolescent, Obesity, overweight, physical activity, sedentarity, adolescent

## Abstract

**Introduction:**

Le but de notre étude était d’évaluer la prévalence du surpoids et de l'obésité chez des adolescents scolarisés dans la ville de Sfax mais aussi, d’étudier son association avec le temps de sédentarité et l'activité physique (AP).

**Méthodes:**

La population étudiée était composée de 1695 adolescents âgés de 15-18 ans. Tous les participants avait rempli un questionnaire porté sur leurs activités physiques et temps de sédentarité, donné lors d'un entretien direct. Le niveau d'AP était évalué avec l'International Physical Activity Questionnaire (IPAQ) version courte.

**Résultats:**

Notre étude comportait 43,7% de garçons et 56,3% de filles. L’âge moyen était de 16,78 ± 1, 1 an. Dans notre échantillon, 23,4% des adolescents étaient en surpoids ou obèses. Le score de l'IPAQ nous a montré que le niveau d'AP de nos participants était faible dans 6,4%, modéré dans 65,4% et élevé dans 28,2% des cas. Nos résultats avaient démontré que l'augmentation du temps de sédentarité (plus de 2 h / jour) est associée à une augmentation significative de l'indice de masse corporelle (IMC) et du tour de taille (TT) (P <0,001). Alors qu'un niveau d'AP élevé et/ou la participation aux séances d'AP structurées dans le cadre scolaire et hors scolaire est accompagnée par une diminution significative de l'IMC et du TT (P <0,001).

**Conclusion:**

Nos résultats apportent une preuve supplémentaire sur la nécessité de promouvoir la vie active chez les jeunes Tunisiens.

## Introduction

La prévalence du surpoids et de l'obésité s'est élevée à un rythme alarmant au cours de ces dernières décennies notamment chez l'enfant et l'adolescent, devenant ainsi l'un des plus grands défis pour la santé publique au 21^ème^ siècle [1, 2]. Les adolescents en surpoids et obèses risquent de rester obèses à l’âge adulte. Ils risquent aussi de développer des maladies métaboliques (diabète) et cardiovasculaires à un âge plus précoce [2, 3]. L'obésité de l'enfant et de l'adolescent est un problème mondial qui affecte aussi bien les pays développés que les pays à revenu faible et intermédiaire, en particulier en milieu urbain [1, 3, 4]. La Tunisie n'est pas épargnée de ce phénomène [5, 6]. En effet cette surcharge pondérale est attribuée essentiellement à un mode de vie qui favorise l'apport excessif d'aliments riches en graisses et en sucre, et qui décourage la pratique de l'activité physique (AP) [1]. Certains facteurs qui influent sur la dépense énergétique, tel le niveau d'AP, explique en grande partie l'apparition de l'obésité chez l'enfant et l'adolescent [7, 8]. Généralement, l'AP diminue avec l’âge en particulier au cours de la période pubertaire et de l'adolescence [9]. L'AP régulière est considérée comme une des pierres angulaires du traitement de la surcharge pondérale, des maladies métaboliques et cardiovasculaires [7, 8, 10]. Alors que la sédentarité ou l'absence d'AP est démontrée comme un facteur de risque d'obésité et de maladies chroniques [8, 10, 11]. Toute fois, cette relation"dose-réponse" entre la sédentarité, le niveau et le type d'AP et son impact sur l'obésité n'est pas encore clarifiée chez les enfants et les adolescents et nécessite d’être vérifiée [10]. Le but de ce travail est d’étudier la prévalence du surpoids et d'obésité ainsi que l’évaluation de la sédentarité et de l'AP chez des adolescents scolarisés en vue d’étudier l'association entre le niveau d'AP et la corpulence de ces jeunes.

## Méthodes

Type de l’étude: il s'agit d'une enquête descriptive, transversale qui a porté sur un échantillon représentatif d'adolescents durant une période de 9 mois allant du mois de Novembre 2012 jusqu'au mois de Mars 2013. Ce travail a été réalisé selon les recommandations de la Déclaration d'Helsinki et a reçu l'approbation de la direction nationale et régionale de la Ministère de l'Education et de Formation.

Population étudiée: l’échantillon était sélectionné au hasard parmi des élèves inscrits dans les classes de 1^ère^, 2^ème^, 3^ème^ et 4^ème^ année secondaire du lycée public (Majida Boulila) de la région de Sfax. La population totale comportait 2264 (998 garçons et 1266 filles) élèves. Nous avons exclu de l’étude les personnes qui ne présentaient aucune motivation à l'adhésion au protocole. Egalement, les élèves pour lesquels les données étaient «incomplètes». La population d’étude était composée de 1695 adolescents âgés de 15 à 18 ans. Un consentement éclairé a été signé par les élèves et leurs parents pour participer à cette étude.

### Recueil des données

Description du protocole: toutes les mesures anthropométriques et le recueil des différentes données ont été réalisés avec la collaboration du médecin scolaire et les enseignants d’éducation physique.

### Mesure des paramètres anthropométriques

Le poids (en kg) était évalué à 0,1 kg près en utilisant une balance manuelle (Seca 709, France), entre 8h du matin et midi chez des sujets déchaussés en tenue légère et avec vessie vide. La taille était mesurée avec une précision de 0,5 cm à l'aide d'une toise fixée au mur, sur des sujets déchaussés, pieds joints bien à plat sur le sol, dos, fesses et talons étaient appliqués contre la planche verticale de la toise et la tête placée en position horizontale de sorte que la ligne de vision soit perpendiculaire au corps. L'indice de masse corporelle (IMC) était calculé en divisant le poids par la taille au carré IMC = Poids/Taille^2^ (kg/m^2^). Pour évaluer le statut pondéral des élèves, nous avons utilisé les données définies par l'International Obesity Task Force (IOTF) [12] et les données de références Françaises [13]. - Le tour de taille (TT) était mesuré en cm avec un mètre à ruban non élastique appliqué, à un point intermédiaire entre la bordure inférieure de la cage thoracique et la crête iliaque et ceci à la fin d'une expiration normale.

### Questionnaire

Nous avons conçu ce questionnaire en deux parties concernant la mesure de la sédentarité d'une part et l'AP d'autre part. Pour plus de fiabilité, ce questionnaire a été donné par les enseignants d’éducation physique ceci lors un entretien avec chaque participant.

### Mesure de la sédentarité

La sédentarité a été approchée, par le temps passé devant un écran (télévision; ordinateur ou jeux vidéo). C'est une mesure efficace et simple pour évaluer le niveau de sédentarité des enfants et des adolescents [11]. Dans notre étude le temps moyen passé dans les activités sédentaires est mesuré pour une semaine habituelle avec jours d’école et de week-end: ((temps en minutes pour un jour d’école x 6) + (temps en minutes pour un jour de week-end)) /7. Aussi, nous avons classé les moyennes trouvées en trois niveaux: inférieur à deux heurs par jour (<2 heures / jour), entre 2et 4 heures par jour et supérieur à 4heurs par jour (> 4h / jour).

### Mesure de l'activité physique

La première partie de notre questionnaire de mesure de l'AP était basée sur la participation ou pas de nos adolescents à des séances d'AP dans le cadre scolaire ou institutionnalisé (club ou association d'activité sportif) hors scolaire. Dans la deuxième partie nous avons utilisé l'International Physical Activity Questionnaire (IPAQ) dans sa version courte [14]. Ce questionnaire, a permis de décrire l'AP des individus de 15 ans et plus, selon l'intensité (marche, intensité modérée, intensité élevée), la fréquence hebdomadaire et la durée journalière de leurs activités physiques au cours des 7 jours qui ont précédé l'entretien. A partir de ces données (intensité, fréquence et durée), les règles préconisées par le groupe IPAQ ont permis d'estimer une dépense énergétique hebdomadaire en équivalents métaboliques (MET), exprimée en MET-minutes/semaine. Le calcul des METs a permis ensuite de classer les individus. Trois catégories IPAQ d'AP sont définies par le groupe IPAQ: élevé, modéré et bas [14, 15].

### Analyse statistique

Les analyses statistiques ont été réalisées sur le logiciel SPSS version 20 (Inc. Chicago, IL, USA). Les moyennes des paramètres ont été comparées à l'aide de tests paramétriques de comparaison de moyennes (test t de Student). Les comparaisons de moyenne et de fréquence ont été effectuées par des tests statistiques de type ANOVA et Chi2, respectivement pour les données quantitatives et qualitatives. Pour les différences significatives, un test post-hoc de comparaison de moyennes, le PLSD de Fisher, a été réalisé. Sauf précision, les données sont exprimées en moyenne ± écart-type ou pourcentage, selon le type de données. Pour tous les tests effectués le seuil de significativité a été fixé à p < 0,05.

## Résultats

Notre population comportait 1695 adolescents dont 741 garçons (43,7%) et 954 filles (56,3%) d’âge moyen 16,78 ±1,1ans.

### Evaluation des paramètres anthropométriques

Les participants avaient tous eu une mesure du poids, de la taille, du TT et un calcul de l ‘IMC. Le Tableau 1 illustre les caractéristiques anthropométriques de notre population selon la corpulence. En effet, le poids moyen de nos participants était 61,52±11,79 kg/m^2^. L'IMC et le tour de taille moyen étaient respectivement de 22,08±3,64 cm et de 71,2±8,54 cm. La moyenne de l'IMC et du TT chez les filles et les garçons était respectivement de (22,24 ± 3,88 vs 21,87±3,43 kg/m^2^; p = 0,04) et (70,01±7,6 vs 72,74±9,41 cm, P < 0,001).

**Tableau 1:** Caractéristiques anthropométriques de la population d’étude selon la corpulence

Taille (cm)	Le groupe total (1695)	Poids normal (1295)	Surpoids ou obèses (400)
Poids (kg)	166,78±8,4 [143, 193]	167,02±8,54 [143, 191]	166,01±8,03 [148, 193]
IMC (kg/m^2^)	61,52±11,79 [30, 139]	57,37± 8,01 [30, 90]	74,91±12,14 [56, 139]
Tour de taille (cm)	22,08±3,64 [14,5, 43,4]	20,53±2,10 [14,5, 4,9]	27,08±3,03 [23,5, 43,4]
Taille (cm)	71,2±8,54 [51, 128]	68,32±5,49 [51, 90]	80,54±9,9 [58, 128]

### Prévalence de l'obésité et du surpoids

**Figure 1:**
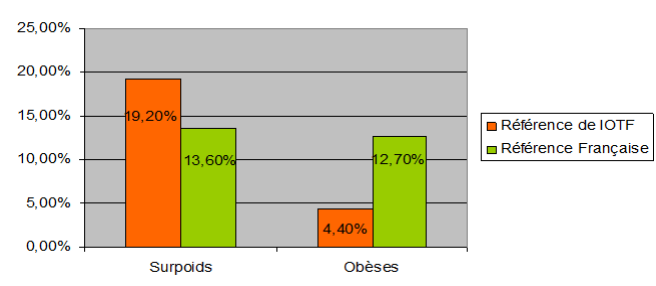
Prévalence du surpoids et d'obésité dans notre population selon les références françaises et de l'IOTF

Figure 1 montre la fréquence de la surcharge pondérale et de l'obésité selon l'ITOF et les courbes françaises. Selon les définitions de l'IOTF, la fréquence de la surcharge pondérale était de 19,2% et celle de l'obésité était de 4,4%, alors que ces fréquences étaient de 13,6% et 12,7% respectivement selon les références Françaises. La distribution du statut pondéral selon le sexe était marquée aussi par une différence significative (p = 0,005). En effet, la prévalence du surpoids était de 21,2% chez les filles et de 16,6% chez les garçons. Cependant la prévalence de l'obésité était de 3,4% chez les filles et de 5,8% chez les garçons. Le Tableau 2 illustre la prévalence de la surcharge pondérale et de l'obésité dans notre population d’étude selon le temps de sédentarité et la participation ou pas à des séances d'AP dans le cadre scolaire et institutionnalisés hors scolaire. Nos résultats avaient montré que la fréquence du surpoids et de l'obésité augmentait significativement avec le temps de sédentarité. De même, le taux de surcharge pondérale était significativement plus élevé chez les élèves qui ne faisaient pas d'AP que ce soit dans le cadre scolaire ou institutionnalisés hors scolaire.

**Tableau 2:** Prévalence du surpoids et d'obésité dans notre population selon le temps de sédentarité et la participation ou pas à des séances d’éducation physique dans le cadre scolaire et d'activités sportives institutionnalisées hors scolaire

	Total n	En surpoids /obésité		En poids normal		p-value
		n	(% )	n	(% )	
**Temps de sédentarité**						
≤2 heures	232	44	(19%)	188	(81%)	0,02
Entre 2 et 4 heures	1121	258	(23%)	863	(77%)	
4 heures	342	98	(28,7%)	244	(71,3%)	
**Education physique dans le cadre scolaire**						
Oui	1600	361	(22,6%)	1239	(77,4%)	0,001
Non	95	39	(41,1%)	56	(58,9%)	
**Activités sportivesinstitutionnalisées hors scolaire**						
Oui	123	6	(4, 9%)	117	(95,1%)	0,00
Non	1572	394	(25,1%)	1178	(74,9%)	

### Activité physique et obésité

**Figure 2:**
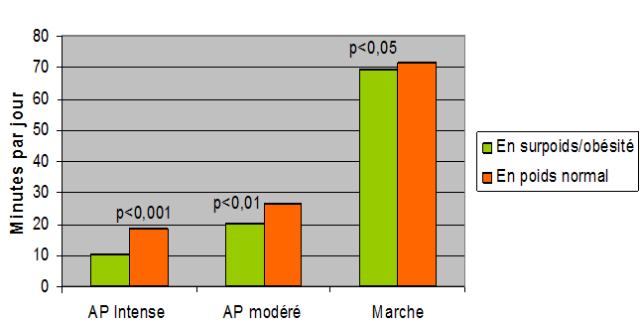
Mesure de l'activité physique de nos participants selon l'IPAQ

Dans notre échantillon seulement 5,6% (8,9% parmi les élèves en surpoids et ou obèses) des élèves ne faisaient pas d'AP au lycée. Alors que seulement 7,2% (1,5% parmi les élèves en surpoids et obèses) étaient inscrits dans un club ou association sportif hors scolaire. Les résultats de l'IPAQ ont démontré que la mesure de l'AP intense de nos sujets était de 16,5± 7,1 min//jour, alors que la mesure de l'AP modéré et de la marche était respectivement de 25,07± 5,1 min/jour et 70,22 ± 13,7 min//jour. La mesure de l'AP de nos participants selon le statut pondérale est représentée dans la Figure 2. Les adolescents avec surcharge pondérale passaient significativement moins de temps dans les différents types d'AP que les sujets de poids normal. Concernant le niveau d'AP de nos participants, selon le score de l'IPAQ, il était faible dans 6,4% des cas, modéré dans 65,4% des cas et élevé dans 28,2% des cas. Le Tableau 3 illustre la relation entre l'AP, la sédentarité et la mesure de l'IMC et du TT. En effet, la participation de nos sujets aux séances d’éducations physiques scolaire et à des activités sportives institutionnalisées hors scolaire était associée une réduction significative de l'IMC et du TT. Egalement, le test ANOVA avait montré une relation statistiquement significative entre le niveau d'activité physique et la corpulence. Dans ce sens, l'IMC et la mesure du TT des élèves faiblement actifs étaient significativement supérieurs à ceux des autres (p < 0,001). En revanche, les tests post hoc avaient montré que la variation de l'IMC et du TT entre les adolescents modérément actifs et actifs n’était pas statistiquement significative (respectivement (p = 0,2) et (p = 0,68)). Nos résultats avait montré également qu'un temps d'activité sédentaire supérieur à 2 h / jour était associé à une augmentation de l'IMC et du TT; (P <0,001). Cependant, les tests post hoc avaient démontré qu'il n'y avait pas de différence significative concernant l'IMC et le TT entre les élèves qui faisaient un temps de sédentarité entre 2 et 4 heures et plus que 4 heures (respectivement (p= 0,2) et (p= 0,13)).

**Tableau 3:** Mesure de l'IMC et du Tour de Taille de notre population en fonction de leurs activités physiques et temps de sédentarités

Variables	IMC (kg/m^2^)		Tour de Taille (cm)	
	Moyenne ± Ecart type	p-value	Moyenne ± Ecart type	p-value
**Activités physiques dans le cadre scolaire**				
Oui	21,98 ± 3,6	0,001	70,97 ± 7,2	0,001
Non	23,72 ± 4,6		75,0 ± 11,5	
**Activités sportivesinstitutionnalisées hors scolaire**				
Oui	20,6 ± 3,4	0,00	69,55 ± 7,3	0,011
Non	22,19 ± 3,6		71,33 ± 8,6	
**Niveau d'AP (IPAQ)**				
Elevé	21,69 ± 3,4	0,00	70,49 ± 7,93	0,00
Modéré	22,02 ± 3,5		70,90 ± 8,10	
Faible	24,38 ± 4,7		77,41 ± 12,35	
**Temps de sédentarité**				
≤2 heures	21,29 ± 3,45	0,00	68,58 ± 7,7	0,00
Entre 2 et 4 heures	22,12 ± 3,47		71,37 ± 8,0	
> 4heures	22,48 ± 4,18		72,43 ± 10,2	

## Discussion

Le nombre d'adolescents qui sont en surpoids ou obèses est en augmentation aussi bien dans les pays à revenu faible qu’à revenu élevé [4]. À notre connaissance, cette étude est la première qui a étudié l'obésité et l'AP chez les adolescents dans la ville de Sfax (Tunisie). Notre enquête est en accord avec d'autres études dans la littérature et qui ont prouvé que la surcharge pondérale chez les jeunes est en augmentation et qu'elle est en relation avec l'augmentation du temps de sédentarité et de la réduction de l'AP [1, 7–9]. Dans notre population plus qu'un adolescent sur cinq était en surcharge pondérale. Ce résultat est concordant avec des études récentes en Tunisie et dans le monde et qui ont démontré que la prévalence du surpoids et de l'obésité chez les adolescents augmentaient de façon spectaculaire, surtout en milieu urbain [1, 3, 4, 16–19]. Une étude Tunisienne menée, auprès des adolescents âgés de 15 à 19 ans a révélé que, la prévalence de surpoids et de l'obésité était respectivement de 17, 4% et 4,1% pour les garçons et 20,7% et 4, 4% pour les filles [18]. Aussi, une autre étude effectuée à Sousse chez des enfants et des adolescents scolarisés, a révélé que la prévalence de la surcharge pondérale était significativement plus élevée chez les filles (16,1%) que chez les garçons (11,6%) ce qui est concordant avec nos résultats. Par contre, la fréquence de l'obésité était de 3,7% chez les filles et de 2,8% chez les garçons alors que dans notre étude l'obésité était plus fréquente chez les garçons [19]. De même, la fréquence de la surcharge pondérale chez l'enfant et l'adolescent dans les pays arabes est aussi élevée [3]. En effet, une récente étude Libanaise a rapporté que 34,8% des enfants et des adolescents, âgés de 6-19 sont en surpoids et que 13,2% sont obèses [20]. À l’échelle mondiale, on estime à 170 millions le nombre des enfants (moins de 18 ans) présentant une surcharge pondérale [21]. Selon un rapport de l'Organisation Mondiale de la Santé (OMS), c'est dans les pays à revenu intermédiaire de la tranche supérieure que la prévalence du surpoids chez les enfants est la plus forte, et dans le groupe des pays à faible revenu que le taux de prévalence est le plus faible. De même la surcharge pondérale, est en augmentation dans la quasi-totalité des pays, mais progresse plus rapidement dans les pays à revenu intermédiaire de la tranche inférieure [22]. Concernant les facteurs favorisants, plusieurs études ont mis en évidence l'existence de trouble de l'hygiène de vie. Ceci comporte essentiellement un déséquilibre qualitatif et quantitatif du régime alimentaire chez la majorité des enfants et adolescents obèses [1, 7, 17, 18, 22–24]. La sédentarité, ou l'absence d'AP, est l'un des facteurs de risque de l'obésité et des maladies cardio-vasculaires [1, 11, 22]. Bien que les avantages d'un mode de vie actif soient bien connus, nos résultats comme d'autres études épidémiologiques ont objectivé une prévalence accrue de la sédentarité chez nos adolescents. Nos résultats ont confirmés une autre fois que plus le temps de sédentarité augmente plus les adolescents sont susceptibles de prendre du poids [11, 17, 23, 24]. La prévalence du surpoids et ou de l'obésité chez nos participants était plus élevée chez les sujets qui dépassaient les 4 heures devant la télé et/ou l'ordinateur. Egalement, nous avons constaté qu'un temps de sédentarité plus de 2heures/jour était associé significativement à une augmentation de l'IMC et du TT. Ce constat, est en concordance avec les recommandations récentes qui suggèrent que le temps de divertissement avec les médias (regarder la télévision ou rester devant l'ordinateur) pour les enfants et les adolescents doit être limité à deux heures par jour [11, 23, 24]. Dans ce cadre, les études ont montré qu'une limitation du temps de la sédentarité, même sans augmentation significative de l'AP pourrait diminuer, les valeurs de l'IMC et du TT [25, 26]. En effet, les données scientifiques, ont démontré que la réduction de la sédentarité dépasse largement les problèmes liés à la surveillance du poids et améliore à la fois le bien-être physique et mental de l'adolescent [11]. Il est donc primordial de lutter précocement contre la sédentarité, pendant les premières années, si l'on veut que les enfants et les jeunes grandissent en bonne santé. Ainsi, l'adoption, très tôt, d'un mode de vie physiquement actif et son maintien pendant toute l'existence contribue à la constitution d'une bonne et solide santé à long terme [11, 24, 25].

La littérature a montré que le cadre scolaire semble être un moment privilégié pour que les jeunes soient actifs [27–29]. En effet, selon, Gordon-Larsen et coll, la participation des adolescents dans les séances d'AP dans le cadre scolaire est associée à une augmentation du temps d'engagement dans les Activités Physiques d'intensité Modérée à Intense (APMI) [29]. En Tunisie, les cours d’éducation physique et sportive (EPS) sont obligatoires. Les élèves du secondaire ont deux heures d'EPS par semaine, à l'exception de ceux qui sont inaptes pour la pratique physique. Dans notre étude la majorité des élèves participaient à ces cours d'EPS. Cependant, c'est une minorité qui était inscrite dans des clubs et associations d'AP et sportives hors scolaire. Selon les résultats de l'IPAQ, la majorité de nos participants étaient modérément actifs et avaient un faible temps d'APMI. Les données de la littérature montrent que les niveaux d'AP ont tendance à être faibles chez les enfants et les adolescents [22, 30–32]. D'après l'OMS, moins d'un adolescent sur quatre se conforme aux lignes directrices recommandées pour l'AP (60 minutes quotidiennes d'APMI) [31]. De même, une étude multicentrique récente auprès d’élèves âgés de 11, 13 et 15 ans dans 35 pays de la Région européenne et d'Amérique du Nord, rapporte que plus des deux tiers des jeunes n'atteignent pas le niveau d'AP actuellement recommandé (60 minutes par jour d'APMI, 5 jours par semaine minimum) [32]. Les publications récentes ont montré que les facteurs environnementaux, sociaux économiques et l'accessibilité, une incidence sur les possibilités d'AP des enfants et des adolescents [30, 32]. Chez nos adolescents, il parait que la marche ou le déplacement actif pour se rendre au lycée constituait la base de leurs AP quotidienne. Alors, que le faible taux d'adhésion aux clubs sportifs et le manque en APMI pourraient être attribué à plusieurs facteurs. D'une part, les parents encouragent leurs enfants, en particulier les filles, à s'engager dans les études plutôt que dans les activités physiques ou les loisirs [33]. D'autre part, l'horaire scolaire, l'accès aux installations, le coût, la qualité des équipements et des installations, et le manque des clubs et des structures sportives sont des facteurs qui ne favorisent pas la pratique de l'AP chez nos jeunes [18, 30]. En effet, l'adhésion des enfants et des adolescents obèses aux AP est spontanément faible et ils présentent une limitation à l'effort dont l'origine est multifactorielle [8, 9, 27]. Egalement, dans notre étude, les sujets en surpoids (obésité incluse) avaient un temps d'engagement en APMI plus bas que les autres. Nos résultats avaient montré aussi l'IMC et le TT moyen étaient moins élevés chez les élèves qui avaient un niveau d'AP quotidien plus élevé et/ou qui participaient à des activités physiques et sportives, soit dans le cadre scolaire ou hors scolaire. Ces résultats sont en accord avec ceux de la littérature qui ont démontré qu'une AP régulière peut engendrer une baisse significative de l'IMC, du TT et donc de la masse grasse viscérale [8, 9, 17]. Ces constations sont extrêmement intéressantes du fait que cette adiposité, notamment viscérale est un facteur de risque majeur des maladies métaboliques et cardio-vasculaires [34]. Des études de pratique clinique ont démontré que le changement de style de vie par la réduction du temps de sédentarité ou par programmes d'AP adaptés ont réduit les risques d'apparition de maladies métaboliques [9, 25, 26]. Finalement, comme toute enquête par questionnaire notre étude a des limites. Tout d'abord, nous n'avons pas tenu compte de l'effet de certains facteurs de risque d'obésité tels que, la situation socio-économique et les habitudes alimentaires. Aussi, l'utilisation d'un questionnaire d'AP se présente comme une limite [35]. En effet, il y'avait des difficultés de se souvenir des AP réalisées sur une semaine et d'identifier le temps et l'intensité d'engagement dans ces activités.

## Conclusion

Nos résultats apportent une preuve supplémentaire sur la nécessité d’établir des stratégies de lutte contre la sédentarité et encourager la pratique de l'AP chez les adolescents de la ville de Sfax. Les messages d’éducation concernant l'alimentation et la pratique d'une AP doivent être diffusés dès l'enfance et tout au long du cursus scolaire. Ceci permettra essentiellement de gagner sur le risque de maladies métaboliques et cardiovasculaires chez les adultes de demain. En fin, il est nécessaire de poursuivre ce travail avec des cohortes plus larges et d'utiliser des outils plus objectifs pour mesurer l'activité physique.
